# Transthyretin Induces Insulin-like Growth Factor I Nuclear Translocation Regulating Its Levels in the Hippocampus

**DOI:** 10.1007/s12035-014-8824-4

**Published:** 2014-08-02

**Authors:** Marta Vieira, João R. Gomes, Maria João Saraiva

**Affiliations:** 1 Molecular Neurobiology Unit, IBMC - Instituto de Biologia Molecular e Celular, Rua do Campo Alegre 823, 4150-180 Porto, Portugal; 2 ICBAS - Instituto de Ciências Biomédicas Abel Salazar, Universidade do Porto, Rua de Jorge Viterbo Ferreira no 228, 4050-313 Porto, Portugal

**Keywords:** TTR, IGF-IR, Transcription, Hippocampus, Nuclear translocation

## Abstract

Transthyretin (TTR) is the carrier protein of thyroxine (T_4_) and binds to retinol-binding protein (RBP)-retinol complex. It is mainly synthesized by both liver and choroid plexuses of the brain. Besides these properties, it has a neuroprotective role in several contexts such as Alzheimer’s disease (AD) and cerebral ischemia. Activation of insulin-like growth factor receptor I (IGF-IR) pathways and increased levels of TTR are associated with absence of neurodegeneration in an AD mouse model. In the present study, we verified that young/adult TTR null mice had decreased levels of IGF-IR in the hippocampus, but not in choroid plexus when compared with wild-type age-matched controls. Moreover, we could also demonstrate that conditional silencing of peripheral TTR did not have any influence in hippocampal IGF-IR levels, indicating that TTR effect on IGF-IR levels is due to TTR mainly synthesized in the choroid plexus. In vitro cellular studies, using NIH3T3 cell line and primary cultured hippocampal neurons, we showed that TTR upregulates IGF-IR at the transcription and translation levels and that is dependent on receptor internalization. Using a GFP-IGF-IR fusion protein, we also found that TTR triggers IGF-IR nuclear translocation in cultured neurons. We could also see an enrichment of IGF-IR in the nuclear fraction, after TTR stimulation in NIH3T3 cells, indicating that IGF-IR regulation, triggered by TTR is induced by nuclear translocation. In summary, the results provide evidence of a new role of TTR as a transcription inducer of IGF-IR in central nervous system (CNS), unveiling a new role in neuroprotection.

## Introduction

Transthyretin (TTR) is a 55,000-Da homotetrameric protein, carrier of thyroxine (T_4_) [1] and associates to the retinol-binding protein (RBP)-retinol complex, both in plasma and the cerebrospinal fluid (CSF) [2]. The TTR-RBP complex is a very stable form of retinol transport, allowing its delivery to cells.

TTR is mainly synthesized by liver and by choroid plexus, being secreted to blood and CSF, respectively [3, 4]. In CSF, TTR represents 25 % of total proteins derived from the choroid plexus [5]. Besides its carrier properties, TTR has also been described as a neuroprotective molecule. TTR prevents Aβ toxicity [6] and modulates Aβ brain levels [7]. The neuroprotective role of TTR is extended to other pathologies besides Alzheimer’s disease (AD); in cerebral ischemia, CSF TTR enhances survival of endangered neurons [8], and, under nerve injury conditions, TTR improves nerve regeneration [9].

Insulin-like growth factors (IGFs) are a family of polypeptides that have important functions in development, cell differentiation, plasticity and survival of the nervous system (reviewed in [10]). Most biological actions of IGF-I are mediated through type I IGF receptor (IGF-IR). IGF-IR is a ubiquitously glycoprotein that consists of two extracellular α-domains and two transmembrane β-domains, linked by disulfide bonds [11–13]. The ligand-binding domain is located in the α-subunit and the tyrosine kinase domain is located in the intracellular region of the β-subunit. Upon ligand binding, two main downstream pathways are activated by IGF-IR, namely MAPK/Ras-Raf-Erk and PI3K/Akt/mTor pathway [14, 15]. Physiological responses to IGF-IR tyrosine kinase activation are diverse and include differentiation, proliferation, protection from apoptosis and neurite outgrowth [15–18].

In an AD mouse model, administration of IGF-I induced clearance of Aβ from the brain, hypothesized to occur through the regulation of Aβ transport proteins such as albumin and TTR [19]. The absence of neurodegeneration in the same mice model was hypothesized to be related to increased TTR levels and activation of growth factors signaling pathways [20]. These findings suggest a strong connection between TTR and IGF-IR. The main objective of this work is to dissect the relation between these molecules, clarifying their relationship at the biological level. For that purpose, in vivo and in vitro studies were performed.

## Materials and Methods

### Animals

The number of mice handled for this research was approved by the Institutional and National General Veterinary Board Ethical Committees according to the National and European Union rules. Three- and 9-month-old TTR wild-type (^+/+^) and TTR null (^−/−^) mice [21], in a 129/svJ background, were obtained from the littermate offspring of heterozygous breeding pairs. The animals were maintained under a 12 h light/dark cycle and fed with regular rodent’s chow and tap water ad libitum. Genotypes were determined from tail-extracted genomic DNA, using primers for the detection of exon 2 of TTR (which is disrupted in TTR^−/−^ by insertion of a neomycin resistance gene) as previously described [21].

### Tissue Processing

Mice were sacrificed with a lethal injection of a premixed solution containing ketamine (75 mg/kg) plus medetomidine (1 mg/kg). Brains were removed from the skull and dissected to isolate hippocampus and choroid plexus (lateral ventricles), and immediately frozen at −80 °C, for biochemical analyses.

### TTR Production and Purification

Recombinant mouse and human TTR were produced in a bacterial expression system using *Escherichia coli* BL21 [22] and purified as previously described [23]. Briefly, after growing the bacteria, the protein was isolated and purified by preparative gel electrophoresis after ion-exchange chromatography. Protein concentration was determined using the Lowry method [24].

### Endotoxin Removal

To remove endotoxin, a polymixin B column (Thermo Scientific) was used. Briefly, the column was regenerated with 1 % sodium deoxycholate (Sigma) and washed with pyrogen-free buffer to remove detergent. Recombinant TTR was applied to the column and incubated during 1 h at room temperature. Aliquots of pyrogen-free buffer were added and the flow-through was collected. Protein concentration was determined by the Bradford method [25].

### NIH3T3 Cell Culture

NIH3T3 cells were grown in Dulbecco’s modified Eagle’s medium (DMEM) supplemented with 10 % inactivated fetal bovine serum (FBS), 100 μg/L streptomycin, 100 U/mL penicillin, 300 μg/mL of L-glutamine and maintained at 37 °C in a humidified incubator of 5 % CO_2_/95 % air. Cells, at 80 % confluency, were serum starved for 2 h, rinsed with phosphate-buffered saline (PBS), and then stimulated with TTR (55 μg/mL) in the presence or absence of α-amanitin (Sigma, 10 μg/mL) during 1 h at 37 °C. For the dynasore (Sigma, 80 μM) experiments, cells were stimulated with TTR (55 μg/mL) in the presence or absence of the drug during 4 h at 37 °C. The inhibitor was pre-incubated 30 min before the TTR stimulus.

### Primary Hippocampal Neuronal Cultures

Primary cultures of mouse hippocampal neurons were prepared from the hippocampus of E18-E19 TTR^−/−^ or TTR^+/+^ mice embryos as previously described [26, 27]. Neuronal cultures were maintained in serum-free neurobasal medium (Gibco Invitrogen), supplemented with B27 (Gibco Invitrogen), glutamate (25 μM), glutamine (0.5 mM), and gentamicin (0.12 mg/mL). Cells were kept at 37 °C in a humidified incubator with 5 % CO_2_/95 % air for 7 days, the time required for maturation of hippocampal neurons [28]. Cells were cultured at a density of 90,000 or 80,000 cells/cm^2^ on poly-D-lysine-coated six-well microplates (MW6) (for western blot and real-time PCR experiments) or glass coverslips (for immunocytochemistry studies), respectively. For the dynasore (Sigma, 80 μM) experiments, cells were stimulated with TTR (55 μg/mL) in the presence or absence of the drug during 4 h at 37 °C.

### Western Blot Analysis

Cultured cells and hippocampus were homogenized in lysis buffer containing 20 mM MOPS, 2 mM EGTA, 5 mM EDTA, 30 mM sodium fluoride, 60 mM β-glycerophosphate, 20 mM sodium pyrophosphate, 1 mM sodium orthovanadate, 1 mM phenylmethylsulphonyl fluoride, 1 % Triton X-100, and 1× protease inhibitors mixture (GE Healthcare). Total protein concentration was determined using the Bradford method. Fifty micrograms of protein were applied and separated by 10 % SDS-PAGE and transferred to a nitrocellulose Hybond-C membrane (GE Healthcare) using a wet system. Membranes were dried, blocked 1 h at room temperature in blocking buffer, 5 % BSA in phosphate-buffered saline Tween-20 (PBST), and then incubated overnight a 4 °C with primary antibodies diluted in blocking buffer, namely rabbit polyclonal IGF-IR (1:1,000; Cell Signaling), β-actin (1:5,000, Sigma), and α-tubulin (1:10,000, Sigma). Membranes were then incubated with antirabbit IgG-HRP (1:10,000; Binding Site) and antimouse IgG-HPR (1:5,000; Binding Site), during 1 h at room temperature. Blots were developed using Immun-Star WesternC Chemiluminescent kit (BioRad) and exposed to ECL Hyperfilm (GE Healthcare). Quantitative analyses were performed using the ImageJ software or ImageLab from Biorad® Laboratories.

### Reverse Transcriptase-Polymerase Chain Reaction

Total RNA was isolated using TRIzol reagent (Invitrogen). First-strand complementary DNA (cDNA) was synthesized using the Superscript II kit (Invitrogen). PCR was performed with the following oligonucleotides to IGF-IR: forward 5′-TCTTGGATGCGGTGTCCAATAAC-3′ and reverse 5′-AGGTTGTGTTGTCGTCCGGTGTG-3′; for mouse β-actin: forward 5′-CTCTTTGATGTCACGCACGATTTC-3′ and reverse 5′-GTGGGCCGCTCTAGGCACCAA-3′.

Ethidium bromide-stained gels were scanned using GENE FLASH syngene bio-imaging equipment. The results were analyzed using the ImageJ software.

### mRNA Semiquantification Through Real-Time PCR

Total RNA was extracted from either 7 days in vitro (7DIV)-cultured hippocampal neurons or NIH3T3 cells using TRIzol Reagent (Invitrogen), as previously described [29]. RNA quality and integrity was assessed using the Experion automated gel-electrophoresis system (Bio-Rad, Portugal), as previously described [29]. Samples showing RNA degradation or contamination by DNA were discarded. RNA concentration was determined using NanoDrop 1000 (Thermo Scientific). The samples were aliquoted and stored at −80 °C until further use. cDNA synthesis was performed using 1 μg of total RNA and the SuperScript® cDNA synthesis (Invitrogen, Portugal), as previously described. Samples were stored at −80 °C until further use.

Oligonucleotides used for IGF-IR real-time PCR were: forward, 5′GTGACTCGGATGGCTTCGTTATC3′ and reverse 5′CTTCATCGCCGCAGACTTTGG3′. 18S RNA was used as reference gene with the following primers: forward, 5′AAATCAGTTATGGTTCCTTTGGTC3′, and reverse, 5′GCTCTAGAATTACCACAGTTATCCAA3′. β-actin was also used as a reference gene with the following primers: forward, 5′CTAAGGCCAACCGTGAAAAG3′, and reverse, 5′ACCAGAGGCATACAGGGACA3′. The annealing temperature was 60 °C.

For gene expression analysis, 1 μL of 1:10 diluted cDNA was added to 10 μL of 2× SYBR Green Master Mix (Bio-Rad) and the final concentration of each primer was 250 nM in 20 μL total volume. The thermocycling reaction was initiated by activation of Taq DNA polymerase by heating at 95 °C during 3 min, followed by 45 cycles of a 15 s denaturation step at 95 °C and a 20 s annealing/elongation step at 60 °C. The fluorescence was measured after the extension step, using the iQ5 Multicolor Real-Time PCR Detection System (Bio-Rad). After the thermocycling reaction, the melting step was performed with slow heating, starting at 55 °C and with a rate of 0.5 °C per 10 s, up to 95 °C, with continuous measurement of fluorescence.

Data analysis was performed using Pfaff method for efficiency correction [30]. Results were normalized with 18S RNA or β-actin as internal reference gene because it showed a stable expression in the conditions tested (compared with other reference genes tested).

### Transfection

The expression vector containing IGF-IR fused with a green fluorescent protein tag (GFP) was kindly provided by Rosemary O’Connor (National University of Ireland, Cork, Ireland) [31]. The cDNA of IGF-IR was fused into the green fluorescent protein (GFP) gene in pEGFP-N1 vector (BD Biosciences Clontech) through EcoRI site. The plasmid sequence of pEGFP-N1-IGF-IR was verified by DNA sequencing reactions. Transfection of cultured hippocampal neurons with GFP-IGF-IR was performed by the calcium phosphate coprecipitation method as previously described with minor modifications [32, 33]. Briefly, 2 μg of plasmid DNA were diluted in Tris-EDTA (TE) pH 7.3 and mixed with HEPES calcium chloride pH 7.2 (2.5 M CaCl_2_, 10 mM HEPES). This DNA/TE/calcium mix was added to a 2× HEPES-buffered saline solution (270 mM NaCl, 10 mM KCl, 1.4 mM Na_2_HPO_4_, 11 mM dextrose, and 42 mM HEPES), pH 7.2. The precipitates were allowed to form for 30 min, with vortex mixing every 5 min, to ensure that the precipitates had similar small sizes. Meanwhile, coverslips with cultured neurons were incubated with cultured conditioned medium with 2 mM of kynurenic acid. The precipitate was added drop wise to each coverslip and incubated at 37 °C, 5 % CO_2_, for 3 h. Cells were then washed with acidic (10 % CO_2_) equilibrated culture medium containing 2 mM kynurenic acid and returned to the 37 °C/5 % CO_2_ incubator for 15 min. Finally, the medium was replaced with the initial culture conditioned medium, and the cells were further incubated in a 37 °C/5 % CO_2_ incubator for 48 h to allow protein expression.

### Immunocytochemistry

Cells were fixed in 4 % sucrose/paraformaldehyde and permeabilized with 0.3 % Triton X-100 in PBS. Neurons were then incubated with 5 % bovine serum albumin (BSA) (Sigma) in PBS + 0.1 % Tween 20, for 1 h at 37 °C, to block nonspecific binding, and incubated with primary antibodies, overnight at 4 °C. Cells were then washed five times with PBS + 0.1 % Tween + 0.5 % BSA and incubated with the appropriate secondary antibodies for 1 h at 37 °C. The coverslips were mounted in a fluorescent mounting medium (DAKO, Denmark) and imaging was performed on a laser scanning Confocal Microscope Leica SP2 AOBS SE, using the 40×/63× oil objective. Primary antibodies used were anti-GFP (1:250, Santa Cruz) and anti-IGF-IR (1:500, Cell Signaling); as secondary antibodies, Alexa Fluor 488 and 594 (1:750, Invitrogen) were employed. The fluorescent dye Hoechst 33342 (0.5 μg/ml, 10″ room temperature) was used to stain nuclei.

### Protein Iodination

TTR was iodinated following the iodogen method [34, 35]. Briefly, to reaction tubes coated with iodogen (Sigma), 100 μl of 0.25 M phosphate buffer and 1 mCi (37 MBq) of Na^125^I (NEN) were added, followed by 10–20 μg protein. The reaction was allowed to proceed in ice bath for 20 min. Labeled protein was separated from free iodide in a 5-ml Sephadex G50 column (Amersham Pharmacia Biotech).

### Radioligand Binding Assays

For binding of ^125^I-TTR to soluble IGF-IR (sIGF-IR) (Sigma), 96-well plates (Maxisorb Nunc, Rochester, NY, USA) were coated with 5 μg/well of sIGF-IR (diluted in 0.1 M carbonate buffer, pH 9.6) overnight at 4 °C. ^125^I-TTR was incubated with the plates alone or with 1-, 10-, 100-, and 500-fold molar excess of unlabeled TTR in binding buffer (0.1 % non-fat dry milk in minimal essential medium [Gibco, Gaithersburg, MD, USA]) for 2 h at 37 °C with gentle shaking. Binding was determined after four washes in ice-cold PBS with 0.05 % Tween 20. Then, 0.1 ml elution buffer (NaCl 0.1 M containing 1 % Nonidet P40) was added for 5 min at 37 °C, and the contents of the wells were aspirated and counted in a gamma counter. Specific binding was defined as that observed with ^125^I labeled protein alone minus ^125^I labeled protein in the presence of the different fold molar excess unlabeled.

Binding of ^125^I-sIGF-IR to TTR immobilized in microtiter wells (5 μg/well) was performed in the presence of 0, 1, 10, and 100 M excess of TTR; 1, 10, and 100 cold sIGF-IR; 1, 10, and 40 μg/mL of anti-TTR IgG (α-TTR; Dako) or anti-IGF-IR IgG (α -IGF-IR, Santa Cruz) or non-immune IgG. Experiments were repeated three independent times, and representative results are shown.

### RNAi Experiment

RNAi for mouse TTR gene silencing was employed as previously described [8]. TTR or control siRNA was formulated into a lipid nanoparticle (LNP) delivery system kindly provided by Alnylam Pharmaceuticals (Boston) [36]. TTR^+/+^ (*n* = 6) were injected in the tail vein with mouse TTR siRNA or with LNP control (LNP alone) at a concentration of 1 mg/kg. Two days after injection, serum was collected and mouse TTR levels were evaluated by Mouse Pre-Albumin ELISA kit (Alpco) to confirm liver TTR gene silencing RNAi as compared with LNP control animals. Fifteen days after injection, a second injection was performed in the same conditions as before. Two days later, animals were sacrificed.

### Nuclear Fractionation Protocol

NIH3T3 cells at 80 % of confluency were serum starved for 2 h, rinsed with PBS and then stimulated with TTR (55 μg/mL) during 1 h at 37 °C. Cells were then scraped on ice with buffer A (10 mM HEPES, 1.5 mM MgCl_2_, 10 mM KCl, 0.5 mM DTT, 0.05 % NP40, pH 7.9, plus cocktail of protease/phosphatase inhibitors) and after 10 min centrifuged for 10 min at 3,000 rpm (4 °C). Supernatant was removed, which contained the cytosol/membrane fraction and the pellet was resuspended in buffer B (5 mM HEPES, 1.5 mM MgCl_2_, 0.2 mM EDTA, 0.5 mM DTT, 26 % glycerol, and pH 7.9), supplemented with 300 mM NaCl. Pellets were homogenized and left 30 min on ice, before 30 min of centrifugation at 24,000 × *g* for 20 min. Supernatant was collected, which contains enriched nuclear proteins. Total protein concentration, in the different fractions, was determined using the Bradford method and 10 μg of protein, of both fractions, were analyzed by Western blot.

### Statistical Analysis

Quantitative data are presented as mean ± SEM. Statistical analysis was carried out using Graphpad Prism 5 software. Differences among groups were analyzed by one-way ANOVA (followed by Bonferroni’s multiple comparison test); comparisons between two groups were made by Student’s *t* test. *P* values of lower than 0.05 were considered significant; ***P < 0.001, **P < 0.01, and *P < 0.05.

## Results

### Hippocampus of TTR^−/−^ Animals Have Decreased Levels of IGF-I Receptor

**Fig. 1:**
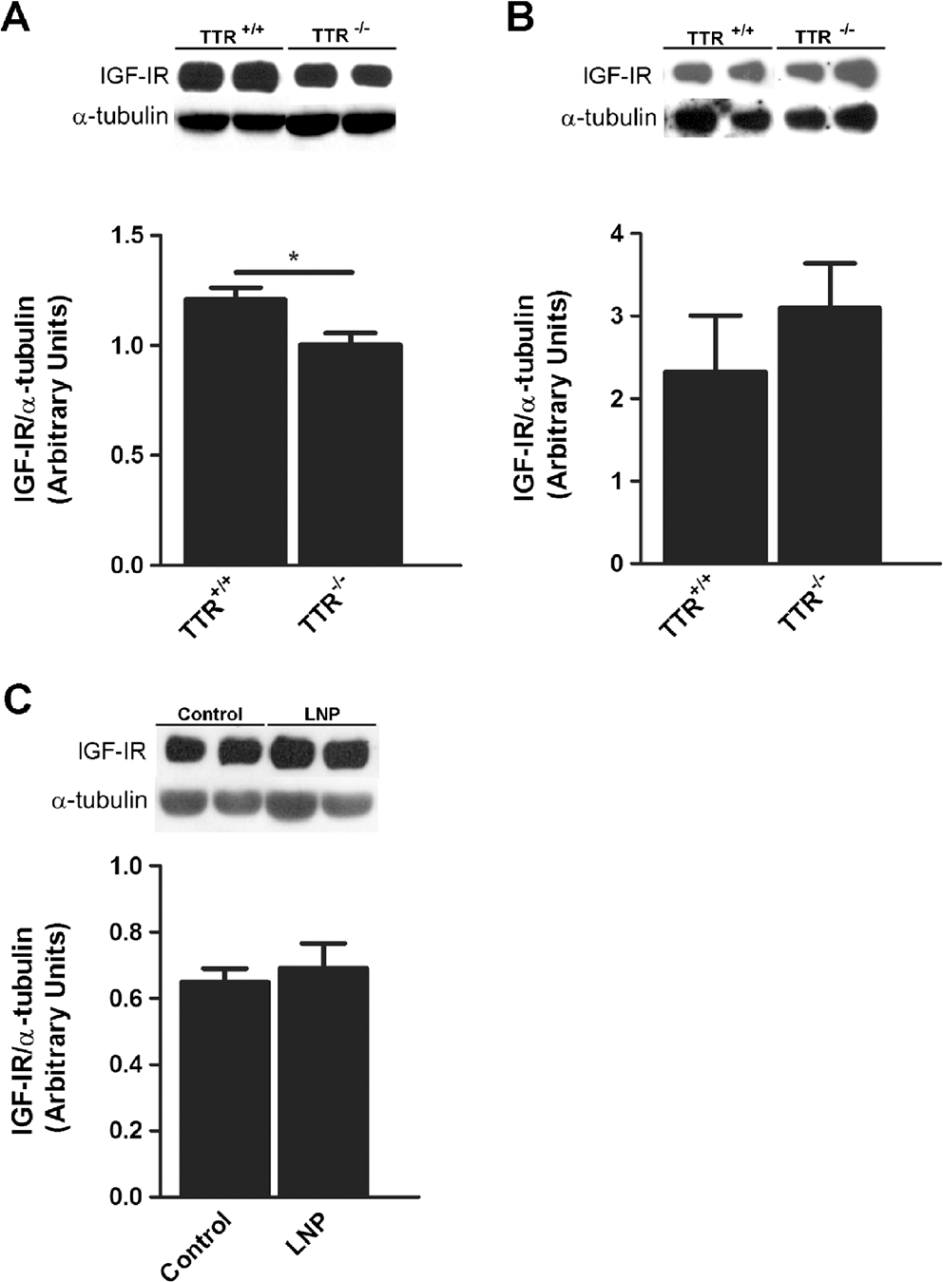
Hippocampus of young TTR^−/−^ mice have decreased levels of IGF-IR. Representative images of western blot analysis and quantitative charts of IGF-IR levels in **a** hippocampus samples of 3 months TTR^+/+^ (*n* = 5) and TTR^−/−^ (*n* = 4) mice; **b** hippocampus samples of 9 months TTR^+/+^ (*n* = 4) and TTR^−/−^ (*n* = 5) animals; **c** hippocampus samples of control (*n* = 6) and LNP (*n* = 6)-treated mice. Results are presented as average ± SEM. *Error bars* represent SEM. *P < 0.05 in a Student’s *t* test

TTR and IGF-IR connection was not clearly understood, but it is known that both molecules were decreased in normal aging. To understand the relationship of TTR and IGF-IR, hippocampus of TTR^−/−^ and TTR^+/+^ mice from different ages, were homogenized and analyzed by western blot to IGF-IR. At 3 months of age, TTR^−/−^ animals had 22 % decreased levels of IGF-IR when compared with age matched wild-type littermates (Fig. 1a) but this difference was abolished in 9-month-old animals (Fig. 1b).

To discern if TTR effect on IGF-IR levels in hippocampus was due to plasma or CSF TTR, the two main sources of TTR in the body, a RNAi experiment was performed. It consisted in the elimination of TTR expression from the liver in young TTR wild-type mice maintaining choroid plexus TTR expression.

TTR levels in plasma decreased approximately 83 % in TTR-LNP-treated animals when compared with LNP control mice, whereas in CSF TTR no differences were found between both groups (data not shown). Abolishment of liver TTR expression did not alter the levels of IGF-IR in the hippocampus, when compared with control animals (Fig. 1c). These results suggest that TTR effect on IGF-IR levels results from CSF TTR action.

### Levels of IGF-IR in Choroid plexuses of TTR^−/−^ Animals are Similar to TTR Wild-Type Littermates

**Fig. 2:**
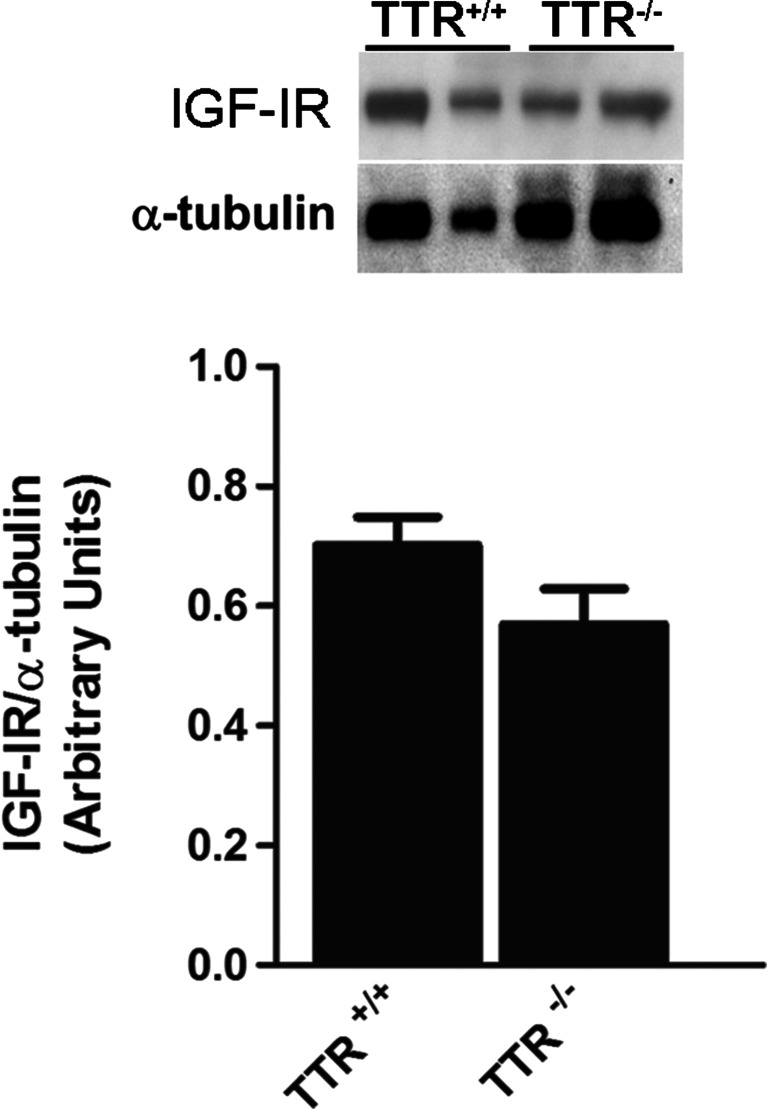
Young TTR null mice have similar levels of IGF-IR in choroid plexuses when compared TTR wild-type littermates. Representative image of western blot analysis and quantitative charts of IGF-IR levels of choroid plexus samples of TTR^+/+^ (*n* = 6) and TTR^−/−^ (*n* = 9) animals at 3 months of age. Results are presented as average ± SEM. *Error bars* represent SEM

Choroid plexus is responsible for TTR synthesis in brain, and also one of the sites where IGF-IR is most abundant [37], so it became relevant to evaluate IGF-IR levels in this tissue. Western blot analysis of choroid plexus of 3 months animals demonstrated that IGF-IR levels were similar between TTR^+/+^ and TTR^−/−^ mice (Fig. 2).

### TTR Regulates IGF-IR at Transcriptional Level

**Fig. 3:**
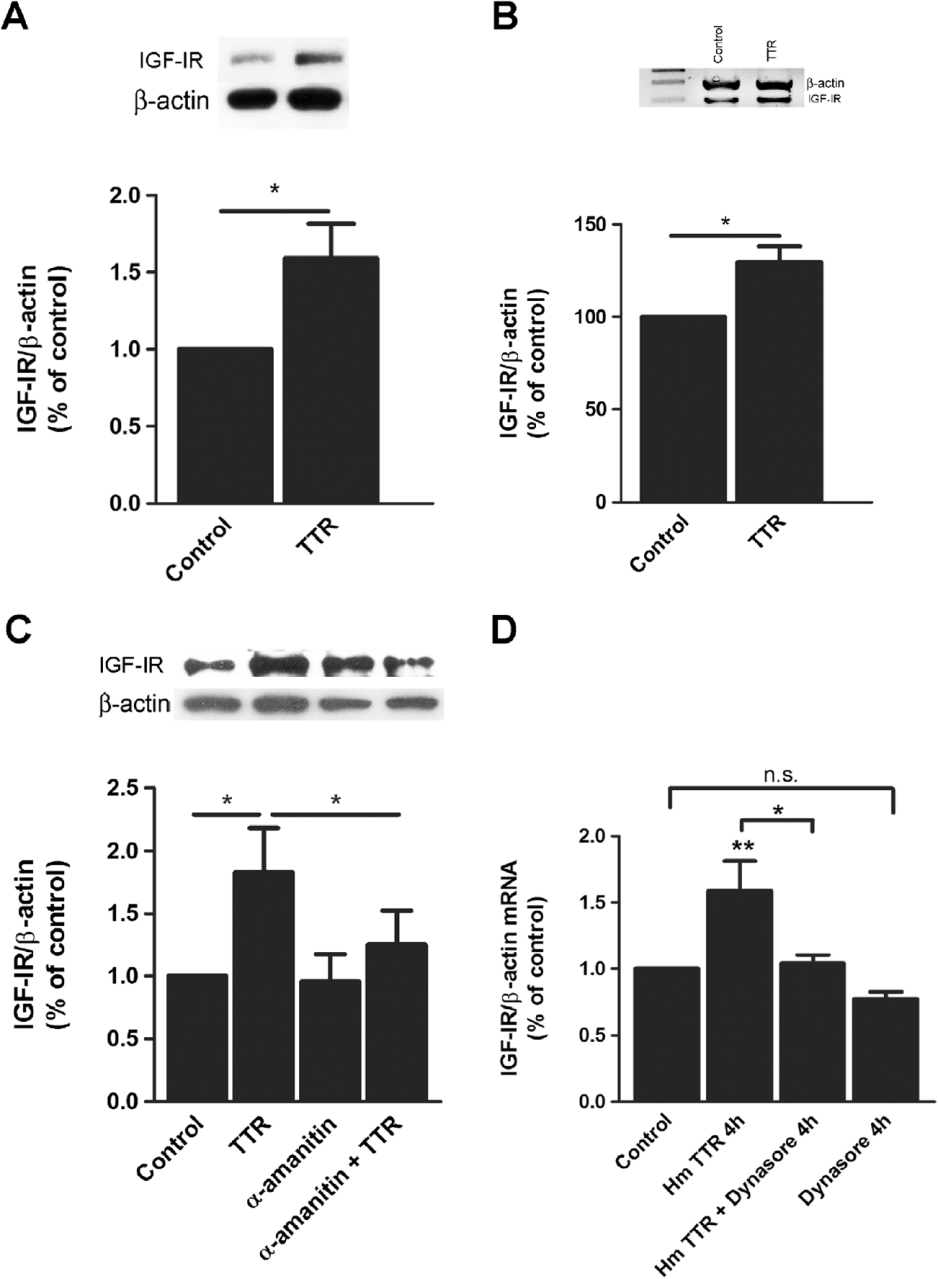
TTR regulates IGF-IR levels. **a** TTR increases IGF-IR protein levels. Representative image and respective chart of western blot analysis of IGF-IR in serum-starved NIH3T3 cells incubated with TTR for 6 h. Data represents the means ± SEM of four independent experiments. **b** TTR increases transcription of IGF-IR. Semiquantitative RT-PCR of serum-starved NIH3T3 cells exposed to TTR during 4 h. Data represents the means ± SEM of three independent experiments. *Error bars* represent SEM. *P < 0.05 in a Student’s *t* test. **c** Western blot analysis of IGF-IR when exposed to TTR for 6 h in the presence or absence of α-amanitin. **d** TTR regulation of IGF-IR levels is dependent on receptor internalization. Total RNA was extracted and IGF-IR and β-actin mRNA were semiquantified through real-time PCR. Data represents the means ± SEM of six independent experiments. Data represents the means ± SEM of five independent experiments. *Error bars* represent SEM. *P < 0.05; in one-way ANOVA, with Bonferroni’s post test

In order to clarify how TTR influenced IGF-IR levels in the hippocampus, cellular studies were performed in NIH3T3 cells. Recombinant endotoxin-free human TTR (55 μg/ml) was added to NIH3T3 cells for 6 h under serum-free conditions. This concentration is under physiological values [38]. Western blot analysis of whole cell extracts showed that in the presence of TTR, IGF-IR levels increased approximately 50 % when compared with controls without added TTR (Fig. 3a).

To discern if the role of TTR occurred at the transcriptional level, semiquantitative RT-PCR of IGF-IR was performed. For that purpose, fibroblasts were incubated with TTR (55 μg/mL) during 4 h under serum-free conditions and RNA was extracted from cells and IGF-IR mRNA quantified by semiquantitative RT-PCR; IGF-IR/β-actin ratios demonstrated that levels of IGF-IR mRNA increased 30 % in cells that had been exposed to TTR when compared with controls (Fig. 3b).

These results suggested that TTR influences transcription of IGF-IR. To confirm this effect, α-amanitin (inhibitor of RNA polymerase, 10 μg/mL) was added to cells during 1 h, before TTR (55 μg/mL) stimulation. After 6 h, cells were lysed and whole cell extract was separated by SDS-PAGE. Western blot analysis showed that when in the presence of α-amanitin, TTR had no longer effect on the regulation of IGF-IR levels (Fig. 3c). Taken together, these results demonstrated that TTR upregulates IGF-IR transcription.

The next step was to understand if this regulation was dependent on receptor endocytosis or just through intracellular signaling pathways. To address that question, we blocked endocytosis, mainly clathrin-mediated endocytosis, through the inhibitor dynasore (80 μM). We saw that the inhibitor blocked the upregulation of IGF-IR mRNA triggered by TTR (Fig. 3d). So this indicates that this effect is dependent on receptor endocytosis.

### TTR Regulates IGF-IR Transcription in Cultured Hippocampal Neurons

**Fig. 4:**
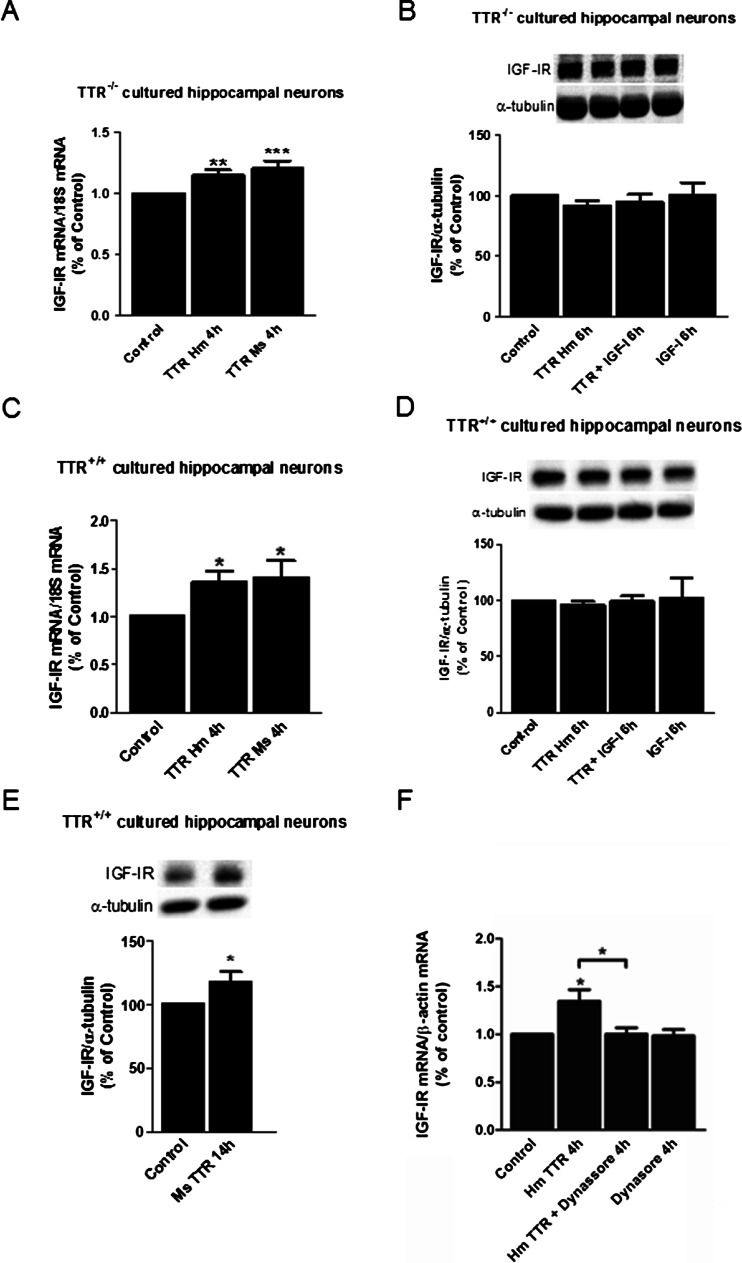
TTR regulates IGF-IR mRNA levels in primary cultured hippocampal neurons, from both wild-type and TTR null mice. Cultured hippocampal neurons from TTR null mice (**a**) and wild-type mice (**c**) were stimulated with mouse (*n* = 3) and human transthyretin (*n* = 4) during 4 h (55 μg/ml), with culture conditioned medium. Total RNA was extracted and IGF-IR and 18S mRNA were semiquantified through real-time PCR. In another set of experiments, cultured hippocampal neurons from TTR null mice (**b**) and wild-type mice (**d**) were stimulated with human TTR (*n* = 3) (55 μg/ml) and/or IGF-I (*n* = 3) (100 ng/ml) during 6 h, with the culture conditioned medium. IGF-IR and tubulin protein levels were determined by western blot. **e** Cultured hippocampal neurons from wild-type mice were stimulated with mouse TTR (*n* = 9) (55 μg/ml) during 14 h, with the culture conditioned medium. IGF-IR and α-tubulin protein levels were determined by Western blot. **f** TTR regulation of IGF-IR levels in cultured hippocampal neurons is dependent on receptor internalization. Total RNA was extracted and IGF-IR and β-actin mRNA were semiquantified through real-time PCR. Data represents the means ± SEM of four independent experiments. Statistical analysis was performed using one-way ANOVA followed by Bonferroni’s multiple comparison tests. *P < 0.05, ***P < 0.001, compared with control; **P < 0.01 for the indicated comparison

Since TTR has been shown to be a neuroprotective molecule, it would be important to know if the observed effects in the NIH3T3 cell line were also present in primary neurons. So we stimulated primary cultured hippocampal neurons, with 7DIV (already mature), with recombinant endotoxin-free human and mouse TTR. In order to avoid possible TTR contamination by choroid plexus epithelia (rich in TTR) or in vitro neuronal TTR production, we used hippocampus of E18-E19 TTR^−/−^ mice embryos to culture hippocampal neurons. After 7DIV, we stimulated the cultures with human or mouse TTR (55 μg/ml) during 4 h. This led to a significant upregulation of IGF-IR mRNA in both instances as determined by semiquantitative real-time PCR (Fig. 4a). At the same time, IGF-IR protein was also analyzed by western blot. We stimulated cultured TTR null hippocampal neurons with human TTR (55 μg/ml) and/or recombinant human IGF (100 ng/ml), for 6 h, since TTR and IGF-I have shown to act synergistically in IGF-IR signaling (submitted manuscript). We could not see any significant change in IGF-IR levels at this time point (Fig. 4b).

To understand if the transcriptional control of IGF-IR by TTR was only restricted to TTR^−/−^ mice or could also be observed in normal wild-type animals, we cultured embryonic hippocampal neurons from wild-type mice and stimulated them with human or mouse TTR (55 μg/ml) during 4 h. An even more robust IGF-IR mRNA upregulation could be seen (Fig. 4c). IGF-IR protein was also analyzed by western blot in these cultures (Fig. 4d); the results were similar to those observed in the TTR^−/−^ cultures (where no endogenous TTR is present, and the observed effect on IGF-IR levels is due to exogenous TTR), i.e., IGF-IR protein levels did not differ with TTR addition to the cultures.

Since IGF-IR is a membrane protein, it is expected to take longer to be delivered from the soma, where it is synthesized, to the dendrites and axons, in neurons. In order to clarify whether TTR only upregulates IGF-IR mRNA and not IGF-IR protein, we stimulated wild-type cultured hippocampal neurons with TTR for longer periods (14 h), in the probability of IGF-IR being a protein of slow turn-over. At this time point, a significant upregulation of IGF-IR protein was observed (Fig. 4e), indicating that TTR upregulates both IGF-IR protein and mRNA.

In cultured hippocampal neurons, we could also show what was observed in NIH3T3 the cell line, i.e., by blocking endocytosis with the inhibitor dynasore, we abrogated the upregulation of IGF-IR mRNA triggered by TTR (Fig. 4f).

### IGF-IR–TTR Interaction

**Fig. 5:**
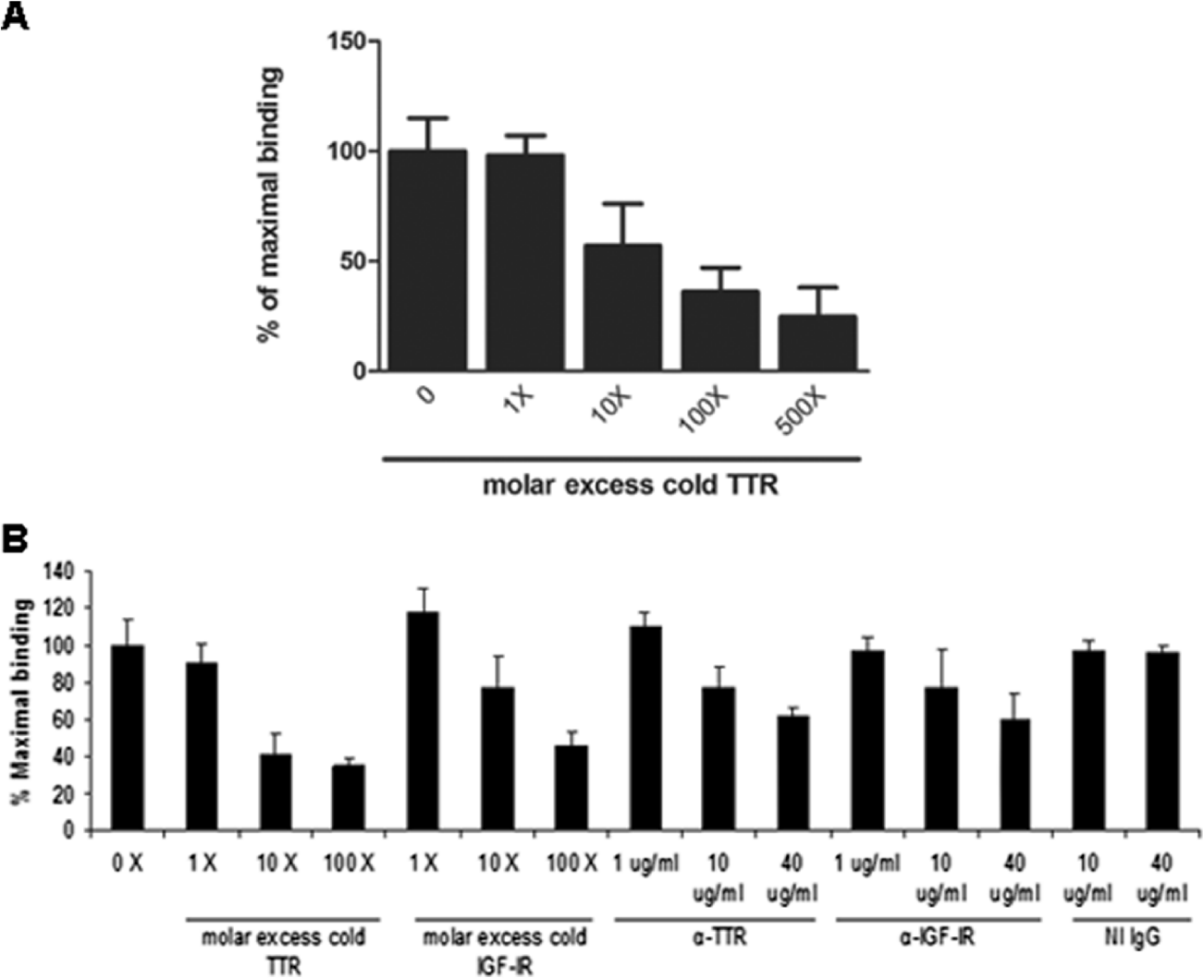
TTR interacts with IGF-IR. Binding of ^125^I-TTR to sIGF-IR immobilized in microtiter wells (5 μg/well). **a** ^125^I-TTR was added to each well in the presence or absence of the indicated molar excess of cold TTR. **b** Binding of ^125^I-sIGF-IR to TTR immobilized in microtiter wells (5 μg/well) in the presence or absence of the indicated molar excess of TTR, cold sIGF-IR , anti-TTR IgG (α-TTR), anti-IGF-IR IgG (α-IGF-IR), or non-immune IgG (NI IgG)

When sIGF-1R was adsorbed to microtiter wells at a constant concentration and incubated with ^125^I–TTR, addition of cold TTR resulted in dose-dependent inhibition of binding (Fig. 5a); if, on the other hand, TTR was immobilized to the plates and incubated with ^125^-sIGF-1R, addition of cold sIGF-IR, TTR, anti-TTR IgG, or anti-IGF-IR IgG resulted in dose-dependent inhibition of binding, whereas non-immune rabbit IgG had no effect (Fig. 5b).

With these results, it seems that TTR interacts with IGF-IR directly and the increased IGF-IR mRNA and protein, induced by TTR, might by due to the nuclear translocation of IGF-IR.

### TTR Induces Migration of IGF-IR to Nucleus

Since IGF-IR has been shown to translocate to the nucleus and to regulate gene expression [39, 40], we hypothesized that TTR interaction with IGF-IR could trigger its translocation to the nucleus where it can stimulate IGF-IR gene expression. So to better visualize the putative IGF-IR translocation, we took advantage of a GFP fusion protein with IGF-IR. The plasmid pEGFP-N1 was used since it fuses GFP to the C-terminal of IGF-IR and avoids any possible blocking of the TTR IGF-IR interaction in the N-terminal. Control immunocytochemistry experiments showed that localization of GFP_IGF-IR is similar to that of the endogenous IGF-IR in cultured hippocampal neurons (data not shown). We also demonstrate that GFP and IGF-IR antibodies recognized the fusion protein as expected.

**Fig. 6:**
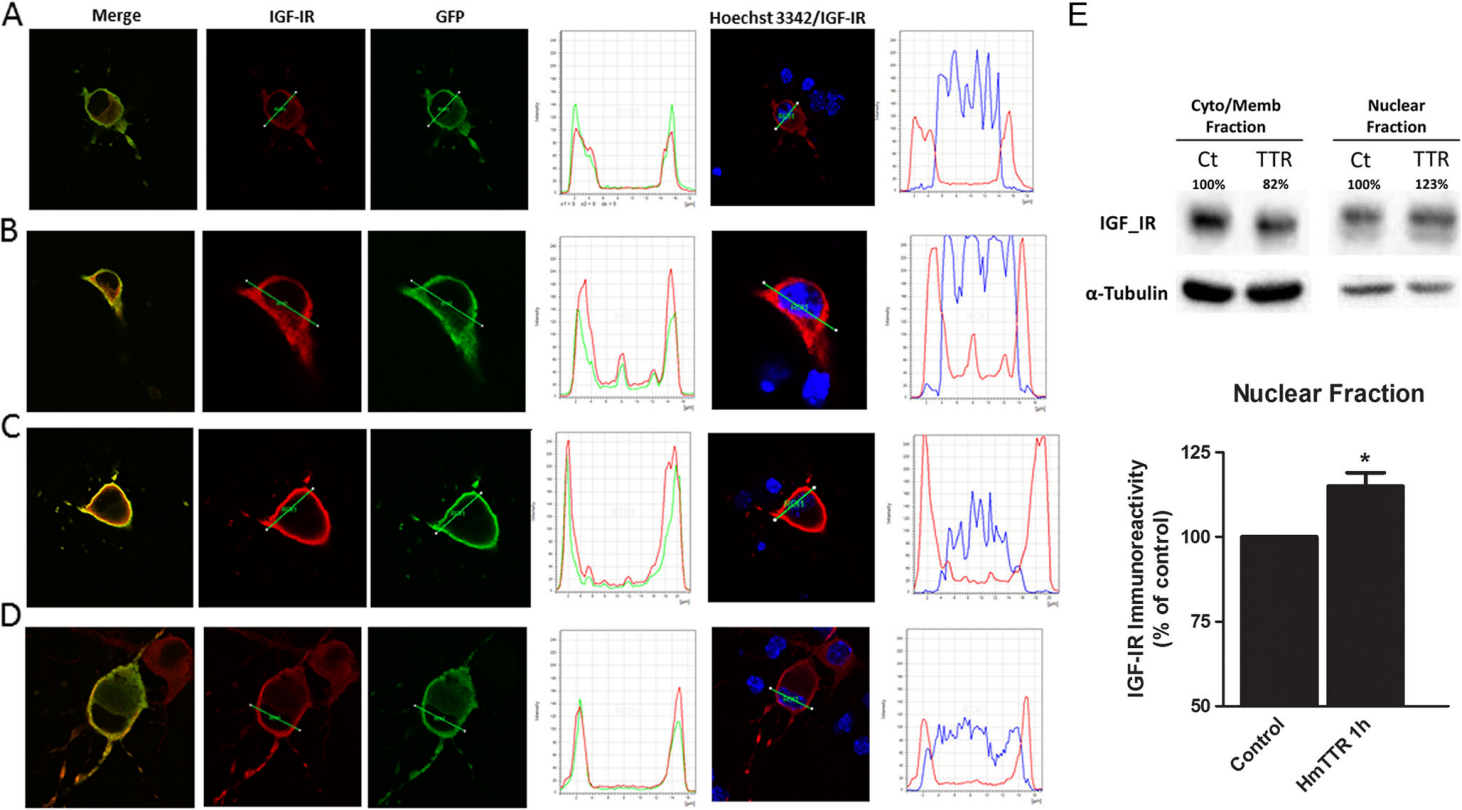
IGF-IR translocates to the nucleus after TTR stimulation. Cultured hippocampal neurons (7DIV) were transfected with GFP-IGF-IR for 48 h (**a** control) and then stimulated with wild-type mouse transthyretin for 1 h (55 μg/mL) (**b**, **c**) or with IGF-I for 1 h (1 μg/mL) (**d**). Cells were then fixed, permeabilized, and incubated with primary antibodies against GFP (*green*), IGF-IR (*red*), and Hoechst 33342 (*blue*). Confocal z-stack images of the transfected GFP-IGF-IR are shown on the left (**a**–**d**) and a transversal selection of the nucleus is shown on the *right*. The pixel intensities for GFP, IGF-IR, and Hoechst 33342 labeled along the stacks are shown in the graphs. Nuclear colocalization with Hoechst of GFP-IGF-IR is only shown with IGF-IR labeling to prevent Hoechst emission contamination of the green (GFP) spectra. Images represent at least four independent experiments. **e** TTR increases the IGF-IR levels in the nuclear fraction of NIH3T3 cells, after stimulus. Western blot analysis of IGF-IR, in the nuclear vs cytosol/membrane fraction (10 μg total protein in both fractions), when exposed to TTR for 1 h. Data represents the means ± SEM of four independent experiments. *Error bars* represent SEM. *P < 0.05 in Student’s *t* test. Representative image illustrates the four independent experiments

To address whether GFP_IGF-IR translocates to the nucleus, different mouse TTR incubation periods were tested in wild-type-cultured hippocampal neurons (7DIV) transfected with GFP_IGF-IR (48 h transfection), from 10 min to 12 h. We could localize GFP_IGF-IR staining in the nucleus from 20 to 60 min with mouse TTR incubation periods (55 μg/ml; Fig. 6b, c). IGF stimulation, either with 100 ng/ml or 1 μg/ml, for the same periods of time had the same results of control experiment, where no GFP_IGF-IR was seen in the nucleus (Fig. 6a, d).

We could also show, using NIH3T3 cells, that 1 h after TTR stimulus, there is an enrichment of IGF-IR protein at the nucleus, through Western blot (Fig. 6e). At the same time, there is a corresponding downregulation of the IGF-IR protein levels at the membrane/cytosol fraction.

## Discussion

This is the first report that describes TTR as a positive regulator of IGF-IR levels in the hippocampus. This finding was demonstrated by studies in TTR null vs wild-type mice and in vitro-cultured cells. We also found that internalization followed by IGF-IR nuclear translocation seems to underline this regulation triggered by TTR.

Analysis of IGF-IR levels in hippocampal samples showed that TTR null mice had decreased levels when compared with TTR wild-type littermates at 3 months of age. Silencing of TTR expression in the liver only did not induce any difference in IGF-IR levels in the hippocampus when compared with nontreated controls. This result suggests that TTR effect on IGF-IR levels in the hippocampus was due to CSF TTR. The presence of TTR in CSF is mainly derived from synthesis and secretion by choroid plexus. In this epithelium, IGF-IR levels were similar between wild-type and TTR null animals; thus, besides molecular differences between cell types, we hypothesized that the difference observed in hippocampal IGF-IR levels between TTR wild-type and TTR null mice is related to the action of exogenous circulating CSF TTR. We excluded the hypothesis of possible TTR synthesis in the hippocampus, since the residual levels that were found by some authors in hippocampus can be attributed to choroid plexus contamination during experimental procedures [41].

Analysis of IGF-IR levels in the hippocampus of 9-month-old animals did not reveal any differences. TTR levels in CSF decrease with age—18 month-old animals had a 30 % reduction in CSF TTR when compared with 5-month-old animals [42]. It is reasonable to speculate that the decrease of TTR could be a factor to abolish difference in IGF-IR levels between TTR wild-type and TTR null mice at this age.

Using NIH3T3 mouse embryonic fibroblast cell line, we started to dissect how TTR regulates IGF-IR. We showed that TTR regulates both transcriptional and translational levels of IGF-IR. To clear whether these results are also relevant in neuronal cells, we used primary cultured hippocampal neurons. TTR upregulation at the transcriptional and translational levels was also seen, not only in TTR null mice but also in wild-type hippocampal neurons, corroborating the notion that the observed TTR effects are described to exogenous TTR and not related to TTR synthesis. This allows us to speculate that TTR action over IGF-IR levels in neurons explains in part some of the neuroprotective properties that have been attributed to TTR in different models of cerebral ischemia [43] or AD [7].

IGF-I decreases IGF-IR mRNA in muscle and neuroblastoma cell lines. The decrease was attributable to transcriptional activity and not due to changes in mRNA stability [44]. On the other hand, in vivo, increased postnatal levels of IGF-I have been associated to lower levels of IGF-IR mRNA [45]. We have analyzed IGF-I levels in plasma and did not find any differences between TTR wild-type and TTR null mice (submitted manuscript), indicating that regulation of IGF-IR levels by TTR is unlikely related to IGF-I levels. Corroborating this, we also observed that IGF-I stimulus in cultured hippocampal neurons did not affect its own receptor levels (Fig. 4b, d), as well as its nuclear translocation (Fig. 6d).

TTR null mice are healthy and fertile, although they present lower levels of plasma retinol and thyroid hormone [21]. In brain, a slight difference on T_4_ levels is observed between TTR wild-type and TTR null mice [46]. Some studies associate retinol and T4 with IGF-IR levels. Retinol deficiency was associated with increased IGF-IR expression in some tissues of Japanese quail [47], whereas administration of retinoic acid upregulates IGF-I receptors in lungs [48]. IGF-IR levels can be regulated by thyroid hormone in the pituitary gland [49] as well as in cardiac tissue [50]. However, in our in vitro experiments, increased IGF-IR protein and mRNA levels were obtained under serum-free conditions and in the presence of recombinant TTR, where no ligands were present, suggesting that upregulation of IGF-IR levels is dependent on TTR and not due to the action, so far described, of the ligands transported by this protein.

Several transcription factors are described as regulators of IGF-IR transcription. Sp1 and E2F1 are examples of transcription factors that are potent transactivators of the *IGF*-*IR* gene [51, 52], whereas breast cancer gene-1 (*BRCA1*), p53 and Wilms’ tumor protein-1 (WT1) are negative regulators of *IGF*-*IR* gene [53]. IGF-IR can also be a transcription factor. Despite the fact of being a transmembrane receptor, IGF-IR can translocate to the nucleus through a clathrin-mediated endocytosis, a process that can be regulated by IGF-I [40, 54]. We demonstrated that upregulation of IGF-IR by TTR was blocked in NIH3T3 and cultured hippocampal neurons when dynasore, an inhibitor of clathrin-mediated endocytosis was present. Using GFP fusion protein with IGF-IR we could demonstrate that IGF-IR can translocate to the nucleus, upon TTR incubation. We could see the receptor in the nucleus between 20 and 60 min after TTR stimulation in cultured hippocampal neurons. In NIH3T3 cells, we could also reinforce this result by showing that 1 h after a TTR stimulus, IGF-IR significantly accumulates in enriched fractions of nucleus vs cytosol/membrane fractions, clearly indicating that TTR regulates IGF-IR levels, through its own nuclear translocation. Stimulus by IGF-I only did not produce the same effect, although it has been described in other cells and models, as above referred to. This could be due to the fact that these cells are tumoral cells with already a lot of IGF-IR in the nucleus in control conditions.

Through binding displacement experiments, we obtained evidence for TTR–IGF-IR binding; the detailed characteristics of the binding need further studies (submitted manuscript).

Apoptotic neuronal cell death is characteristic of neurodegenerative disorders, and the IGF-IR role is becoming more relevant to protect from apoptosis [55, 56]. Increased IGF-IR levels induced by TTR, described here for the first time, is an important finding that might be very useful to control IGF-IR levels in many different pathological situations, unraveling possible mechanistic roles of how TTR can be neuroprotective. It is also of the upmost importance search of TTR as inducer of IGF-IR signaling pathways (submitted manuscript).
